# The health informatics cohort enhancement project (HICE): using routinely collected primary care data to identify people with a lifetime diagnosis of psychotic disorder

**DOI:** 10.1186/1756-0500-5-95

**Published:** 2012-02-14

**Authors:** Alexis Economou, Michelle Grey, Joanna McGregor, Nick Craddock, Ronan A Lyons, Michael J Owen, Vaughn Price, Sue Thomson, James TR Walters, Keith Lloyd

**Affiliations:** 1 Centre for Health Information Research & Evaluation, ILS2, College of Medicine, Swansea University, Swansea, SA2 8PP, UK; 2 MRC Centre for Neuropsychiatric Genetics and Genomics and Neuroscience and Mental Health Research Institute, Cardiff University, Heath Park, Cardiff CF14 4XN, UK

## Abstract

**Background:**

We have previously demonstrated that routinely collected primary care data can be used to identify potential participants for trials in depression [1]. Here we demonstrate how patients with psychotic disorders can be identified from primary care records for potential inclusion in a cohort study. We discuss the strengths and limitations of this approach; assess its potential value and report challenges encountered.

**Methods:**

We designed an algorithm with which we searched for patients with a lifetime diagnosis of psychotic disorders within the Secure Anonymised Information Linkage (SAIL) database of routinely collected health data. The algorithm was validated against the "gold standard" of a well established operational criteria checklist for psychotic and affective illness (OPCRIT). Case notes of 100 patients from a community mental health team (CMHT) in Swansea were studied of whom 80 had matched GP records.

**Results:**

The algorithm had favourable test characteristics, with a very good ability to detect patients with psychotic disorders (sensitivity > 0.7) and an excellent ability not to falsely identify patients with psychotic disorders (specificity > 0.9).

**Conclusions:**

With certain limitations our algorithm can be used to search the general practice data and reliably identify patients with psychotic disorders. This may be useful in identifying candidates for potential inclusion in cohort studies.

## Background

The expanding area of health informatics looks to make the best use of the rich sources of clinical information housed in electronic databases. In the area of mental health, recruitment to trials and cohorts can be particularly challenging, but we have previously shown that routinely collected, digitally stored, clinical data from primary care can be used to identify potential participants for trials in depression [1]. We now seek to extend this technique to psychiatric cohort studies by identifying patients with psychotic disorders. The design of an electronic cohort of patients with psychotic disorders in tandem with a traditional cohort of patients could lead to more powerful longitudinal studies and a more complete study of the aetiology, prognostic indicators and treatment response of psychotic disorders.

### Aims

To create an algorithm which can be used to search electronic databases of routinely collected primary care clinical data [2,3];

To examine the algorithm's ability to correctly identify patients with psychotic disorders compared to the 'gold standard' diagnosis generated by OPCRIT [4]; and

To determine whether anonymised routinely collected primary care data can be used to accurately identify patients with psychotic disorders for participation in a cohort study.

## Methods

### Patient selection

The patient sample was taken from the population of a Community Mental Health Team (CMHT) in Swansea. JM generated a list of 200 random numbers, in a range of 1 to 500, using SPSS software. The random numbers were used by ST, VP and AE to select individual paper case notes, for inclusion in the study.

### Data anonymisation

The SAIL database is run by the Health Informatics Research Unit (HIRU) at Swansea University [1]. HIRU has a protocol in place with National Health Service Wales Informatics Service (NWIS) to ensure that all data are anonymised. This has been achieved through a split file approach to data management. The demographic data are separated from the clinical data by the source organisation and a system linking field is used to ensure that the data can be rejoined later. The demographic data are sent to NWIS and the clinical data are sent to HIRU. NWIS use encryption technology for pseudo-anonymisation, replacing the personal data in each record with an Anonymous Linking Field (ALF). This product is then transferred to HIRU where it is joined to the clinical data via the system linking field. As a final safeguard, HIRU further encrypts the ALF, thus ensuring that no single organisation can decrypt the records. This split file method ensures that anonymity and confidentiality is maintained, whilst maintaining the facility of data linkage at the individual level. The data are then ready for research applications. Only the source organisation (i.e. the treating physician) has access to both personal and clinical data. The data are provided to the SAIL database on the grounds that they are never de-anonymised and therefore patient records can never be traced back to individual patients [3]. SAIL is a growing databank of linked data used to support research. It currently contains anonymised GP data on about a million people from 150 practices. The OPCRIT data were anonymised and linked to GP data.

### Ethical approval

The SAIL project conforms to the HIRU Data Anonymisation Policy and Process (DAPP), which takes account of the requirements of the Data Protection Act (1998), the Principles of the Caldicott report (1997) and measures that embody good information governance. The DAPP has been endorsed by Informing Healthcare and the Corporate Health Information Programme (CHIP) and has been reviewed by Caldicott Guardians and Information Governance Officers in the NHS and Local Government. The HICE project was exempted from further ethical approval by South West Wales Research Ethics Committee in July 2008.

### Algorithm construction

The Quality and Outcomes Framework (QOF) rewards primary care practises in England and Wales for the provision of quality care. Payment for meeting QoF targets is dependent on accurate data capture using Read **codes **which are the standard clinical terminology system used in UK primary care. The data returned by general practices in Wales as part of the QoF exercise is included in the datasets that NWIS anonymises and makes available to SAIL. Thus no additional effort is required on the part of general practitioners to generate data that can be used to identify potential cohort participants and anonymity is maintained with only the treating clinical team being able to identify the patient. Once loaded into relational databases such as SAIL it is possible to interrogate this data using an algorithm written in a data handling language called Structured Query Language (SQL) [5]. The algorithm was used to identify Read codes Version 2 (5-byte set) from the General Practice dataset in SAIL by searching for QOF Read codes for psychotic disorders as listed in Table 1[6].

**Table 1:** Quality Outcomes Framework read codes used for diagnosis by General Practitioners

QOF read code	Diagnosis
E10%	Schizophrenic disorders
E110%	Manic disorder, single episode
E111%	Recurrent manic episodes
E1124	Single major depressive episode, severe, with psychotic disorders
E1134	Recurrent major depressive episodes, severe, with psychotic disorders
E114%	Bipolar affective disorder, currently manic
E115%	Bipolar affective disorder, currently depressed
E116%	Mixed bipolar affective disorder
E117%	Unspecified bipolar affective disorder
E11y.	Other and unspecified manic-depressive psychoses
E11y0	Unspecified manic-depressive psychoses
E11y1	Atypical manic disorder
E11y3	Other mixed manic-depressive psychoses
E11yz	Other and unspecified manic-depressive psychoses NOS
E11z.	Other and unspecified affective psychoses
E11z0	Unspecified affective psychoses NOS
E11zz	Other affective psychotic disorders NOS
E12%	Paranoid states
E13..	Other nonorganic psychoses
E130.	Reactive depressive psychotic disorders
E131.	Acute hysterical psychotic disorders
E132.	Reactive confusion
E133.	Acute paranoid reaction
E134.	Psychogenic paranoid psychotic disorders
E13y.	Other reactive psychoses
E13y0	Psychogenic stupor
E13y1	Brief reactive psychotic disorders
E13yz	Other reactive psychoses NOS
E13z.	Nonorganic psychotic disorders NOS
E2122	Schizotypal personality
Eu2%	[X]Schizophrenia, schizotypal and delusional disorders
Eu30%	[X]Manic episode
Eu31%	[X]Bipolar affective disorder
Eu323	Severe depressive episode with psychotic symptoms
Eu333	[X] Recurrent depressive disorder, current episode severe with psychotic symptoms

[X] = External causes of morbidity and mortality

*NOS *Not Otherwise Specified

### Validation

The operational criteria checklist for psychotic and affective illness (OPCRIT) was used to provide a 'gold standard' diagnosis for the 100 patients whose case notes were examined. OPCRIT is a diagnostic system which comprises a checklist of 90 items, constructed from operational criteria for the major psychiatric classifications and a suite of computer programmes that allows data to be entered from patients' case notes. Once the data have been loaded into OPCRIT, diagnoses are generated according to different classification systems [4].

For the purposes of this study, a patient was considered to be suffering with psychotic disorders if the OPCRIT diagnosis corresponded to any of the International Classification of Diseases 10^th ^Revision (ICD 10) codes in Table 2.

**Table 2:** International Classification of Diseases 10^th ^Revision codes used for OPCRIT diagnosis

ICD--10 code	Diagnosis
F20	Schizophrenia
F21	Schizo-typal disorder
F22	Persistent delusional disorders
F23	Acute and transient psychotic disorders
F24	Induced delusional disorder
F25	Schizoaffective disorder
F28	Other non-organic psychotic disorders
F29	Unspecified non-organic psychotic disorders
F30	Manic episode
F31	Bipolar affective disorder
F32.3	Severe depressive episode with psychotic symptoms
F33.3	Recurrent depressive disorder, current episode severe with psychotic symptoms
F34	Persistent mood [affective] disorders
F38	Other mood [affective] disorders
F39	Unspecified mood [affective] disorder

In order to check the reliability of the algorithm in identifying patients with psychotic disorders, the diagnoses generated by OPCRIT in our patient sample were compared with the diagnoses produced by running the algorithm for the same group of patients in the SAIL database.

### Statistical analysis

The data were analysed using The Statistical Package for the Social Sciences (SPSS) version 19. In assessing the reliability of the algorithm the characteristics assessed were: sensitivity (true positive rate), specificity (true negative rate), prevalence (pre-test likelihood of disease), predictive value of positive test (post-test likelihood of disease), and predictive values of negative test (post-test likelihood of no disease), likelihood ratio of a positive result, likelihood ratio of a negative result and the diagnostic odds ratio.

## Results

Out of 100 patients, whose paper case notes were assessed using OPCRIT, 51 met ICD-10 criteria for psychotic disorders. Of these fifty-one patients who met ICD-10 criteria for psychotic disorders, twenty-one were diagnosed with schizophrenia, five with schizoaffective disorder, six with bipolar affective disorder, three with persistent delusional disorder, two with severe depressive episode with psychotic disorders, two with manic episode and twelve patients were diagnosed with other non organic psychotic disorder (Table 3).

**Table 3:** OPCRIT results for 51 patients with psychotic disorders

Diagnosis	%	
Schizophrenia	41.2	(n = 21)
Schizoaffective disorder	9.8	(n = 5)
Bipolar affective disorder	11.8	(n = 6)
Persistent delusional disorder	5.9	(n = 3)
Severe depressive episode with psychotic disorders	3.9	(n = 2)
Manic episode	3.9	(n = 2)
Other non organic psychotic disorder	23.5	(n = 12)

Of the remaining 49 patients, 33 met ICD-10 criteria for non-psychotic mental disorders; the remaining 16 had insufficient clinical information in their case notes to complete all 90 items in OPCRIT in order to generate a diagnosis. These 16 were omitted from the analysis.

Clinical information was stored in the general practice database (GPDB) in SAIL for 80 of the above 100 patients. The 20 patients who belonged to practices that were not currently supplying SAIL with data were omitted from the analysis.

The sensitivity and specificity were calculated only on those cases where there was sufficient information from both OPCRIT and the GP data to make a comparison between the diagnoses (Table 4).

**Table 4:** Two by Two table comparing diagnosis of psychotic data using algorithm derived from General Practice Data compared to gold standard

Psychotic disorder diagnosis	OPCRIT--Yes	OPCRIT--No	Totals
GP data--Yes	33	1	34

GP data--No	9	26	35

Totals	42	27	69

The algorithm derived diagnosis in GP data as a diagnostic test was compared against the gold standard of OPCRIT diagnosis. The test characteristics are shown in Table 5.

**Table 5:** Health Informatics Cohort Enhancement (HICE) Algorithm characteristics

Test Characteristic	Value (95% Confidence Interval)
Sensitivity	0.79 (0.6319-0.8970)
Specificity	0.96 (0.8103-0.9991)
Prevalence	0.61 (0.4837-0.7240)
Predictive value of positive test	0.97 (0.8467-0.9993)
Predictive value of negative test	0.74 (0.5674-0.8751)
Likelihood ratio of positive test	21.21 (3.0798-146.1268)
Likelihood ratio of negative test	4.49 (2.5065-8.0568)
Diagnostic odds ratio	95.33 (11.3410-801.3784)

Further analysis was undertaken to investigate the reasons for the incorrect cases.

One false positive was identified as the patient had a QOF psychotic disorders code in their GP data along with a number of other mental health diagnoses. In the false negative group, none had a psychotic disorders code of any description.

## Discussion

In this study, we built an algorithm and subsequently examined its performance in identifying patients with psychotic disorders, by searching primary care data.

### Main findings

We were able to construct an algorithm to search electronic databases of routinely collected primary care clinical data. The algorithm had very promising characteristics when evaluated against the 'gold standard' of OPCRIT diagnosis. It combined a very good ability to detect patients with psychotic disorders (true positives), with an excellent ability not to incorrectly identify patients who do not have psychotic disorders (true negatives). The other test characteristics included an excellent ability to minimise the number of patients without psychotic disorders who tested positive (false positives) and a very good ability to minimise the number of patients identified as not having psychotic disorders when in fact they did (false negatives). The study suggests that routinely collected primary care data can be used to accurately identify patients with psychotic disorders for participation in a cohort study

### Comparison with previous research

Previous research has demonstrated that general practitioners accurately document psychotic illness in their computer records and that general practice computer records are reliable for research purposes [7,8]. We have previously shown that that routinely collected data in primary care can be used to identify patients suffering with depression for potential inclusion in a clinical trial and described how that data can then be de-anonymised by the treating team without compromising patient confidentiality [1]. The present study demonstrates that an electronic algorithm built to search databanks of clinical information, entered by general practitioners during patient consultations, performs well in identifying patients with a lifetime diagnosis of a psychotic disorder.

### Study limitations

Twenty out of the original 100 patients whose case notes were assessed using OPCRIT did not have clinical information stored in the GP data within SAIL, as they were registered to practices who were not currently supplying data to SAIL, limiting the precision of findings

The algorithm used Quality and Outcome Framework (QOF) Read codes used by general practitioners to document a diagnosis of psychotic disorders. The QOF list of read codes for psychotic disorders appears to be fairly comprehensive; all that are omitted are organic psychoses, psychotic disorders with origins in childhood, seasonal affective disorder, rebound mood swings and some depression codes. Codes that explicitly state depression with psychotic symptoms were included in the QOF. A more modified algorithm could have identified patients with further Read codes, including those regarding prescription of psychotropic medication used in the treatment of patients suffering with psychotic disorders, such as antipsychotics. Of course, antipsychotic medication is prescribed for a variety of clinical presentations and not only for patients with psychotic disorders. Such an alteration to the algorithm would likely have increased the ability to identify patients with psychotic disorders (improved sensitivity) at the expense of perhaps falsely identify patients as having psychotic disorders (reduced specificity) when in fact they had been prescribed psychotropic medication for treatment of clinical presentations other than psychotic disorders. In this event, the diagnostic test would have increased sensitivity but also reduced specificity, as well as reduced positive predictive value. The possibility that OPCRIT diagnosis may be sub-optimal and hence not a gold standard must also be considered. 16 out of 100 paper case notes examined did not include enough clinical information for all items in OPCRIT to be completed. However, this is a limitation inherent in comprehensiveness of clinical notes rather than a limitation of OPCRIT. We also acknowledge that the prevalence of psychosis in the CMHT population is higher than in the community population and this may impact upon the positive predictive value of our algorithm. Thus, further research is needed.

## Conclusions

The algorithm designed to search routinely collected primary data in UK primary care databases PDB can reliably be used to identify patients with psychotic disorders. This will enable researchers to easily identify a large number of patients with psychotic disorders and may be an important tool in trial recruitment. It is also a promising development in the efforts to create population based electronic cohort of patients with psychotic disorders. Further research is needed to test this approach in other disorders.

## Abbreviations

ALF: Anonymous linking field; CHIP: Corporate health information programme; CHIRAL: Centre for health information research and evaluation; CMHT: Community mental health team; DAPP: Data anonymisation policy and process; GPDB: General practice database; HIRU: Health informatics research unit; ICD10: International classification of diseases 10th revision (ICD 10); NISCHR: National institute for social care and health research; NWIS: National health service wales informatics service; OPCRIT: Operational criteria checklist for psychotic and affective illness; QOF: Quality and outcomes framework; SAIL: Secure anonymised information linkage; SPSS: The statistical package for the social sciences; SQL: Structured query language.

## Competing interests

The authors declare that they have no competing interests.

## Authors' contributions

KL conceived the study and oversaw its design coordination and analysis. RL MO, NC and JW assisted. AE carried out the data collection assisted by ST and VP and performed the statistical analysis. JM designed the algorithm. MG provided technical assistance. AE, MG, JM RL, MO, JW NC and KL drafted the manuscript. All authors read and approved the final manuscript.
